# Comparison of DNA-, PMA-, and RNA-based 16S rRNA Illumina sequencing for detection of live bacteria in water

**DOI:** 10.1038/s41598-017-02516-3

**Published:** 2017-07-18

**Authors:** Ru Li, Hein Min Tun, Musarrat Jahan, Zhengxiao Zhang, Ayush Kumar, W. G. Dilantha Fernando, Annemieke Farenhorst, Ehsan Khafipour

**Affiliations:** 1 Department of Soil Science, University of Manitoba, Winnipeg, MB R3T 2N2 Canada; 2 Department of Animal Science, University of Manitoba, Winnipeg, MB R3T 2N2 Canada; 3 Department of Medical Microbiology, University of Manitoba, Winnipeg, MB R3T 2N2 Canada; 4 Department of Microbiology, University of Manitoba, Winnipeg, MB R3T 2N2 Canada; 5 Department of Plant Science, University of Manitoba, Winnipeg, MB R3T 2N2 Canada; 6 Present Address: Department of plant protection, Yunnan Agricultural University, Kunming, Yunnan province 650201 China; 7 Present Address: Department of Pediatrics, University of Alberta, AB, Canada

## Abstract

The limitation of 16S rRNA gene sequencing (DNA-based) for microbial community analyses in water is the inability to differentiate live (dormant cells as well as growing or non-growing metabolically active cells) and dead cells, which can lead to false positive results in the absence of live microbes. Propidium-monoazide (PMA) has been used to selectively remove DNA from dead cells during downstream sequencing process. In comparison, 16S rRNA sequencing (RNA-based) can target live microbial cells in water as both dormant and metabolically active cells produce rRNA. The objective of this study was to compare the efficiency and sensitivity﻿ of﻿ DNA-based, PMA-based and RNA-based 16S rRNA Illumina sequencing methodologies for live bacteria detection in water samples experimentally spiked with different combination of bacteria (2 gram-negative and 2 gram-positive/acid fast species either all live, all dead, or combinations of live and dead species) or obtained from different sources (First Nation community drinking water; city of Winnipeg tap water; water from Red River, Manitoba, Canada). The RNA-based method, while was superior for detection of live bacterial cells still identified a number of 16S rRNA targets in samples spiked with dead cells. In environmental water samples, the DNA- and PMA-based approaches perhaps overestimated the richness of microbial community compared to RNA-based method. Our results suggest that the RNA-based sequencing was superior to DNA- and PMA-based methods in detecting live bacterial cells in water.

## Introduction

Water-borne pathogens and their associated diseases pose a high risk to public health^1^. The majority of research in this area initially focused on fecal-associated pathogens, but in recent years the research has broadened considerably towards non-fecal opportunistic pathogens, such as several species within genera *Legionella* and *Mycobacteria*^2, 3^. Culture-based methods have long been used to detect microbial pathogens in the environment; however, these methodologies are still labor intensive, lengthy, and are not able to detect difficult to cultivate species. As such, only less than 1% of the microorganisms present in the environment, such as in natural water and soil samples, have been cultured with the currently known methods^4^. Although development of culturomic techniques in recent years has helped to culture not previously cultivated organisms that are corresponding to a sequence(s) obtained from a mixed microbial community using 16S rRNA or shotgun whole genome sequencing approaches^5^, the methodology is yet far from being perfect, and thus, sequencing approaches are still the most reliable tool for characterization of members of a microbial community. That being said, DNA-based identification methods do not discriminate between DNA from live (dormant cells as well as growing or non-growing metabolically active cells) and dead microbial cells. This becomes problematic as DNA of dead bacterial cells can persist in the environment depending on the environmental conditions^6^. For instance, DNA of dead bacterial cells has been shown to persist for 25 days in stream water^7^ and 70 days in soil^8^. Considering the slow decay rate of DNA from dead bacterial cells, the DNA-based detection methods have a tendency to overestimate bacterial richness and abundance in a sample; hence, leading to false positive results of live pathogens making them less suitable tool for water quality check.

Specialized methods to detect live bacterial cells are becoming popular. This includes the use of propidium monoazide (PMA), a DNA-intercalating agent that only penetrates membrane-compromised/dead bacterial cells and forms photo-induced crosslinks after exposure to light to allow for the selective removal of dead cells from downstream DNA applications^9–11^. The PMA in combination with real-time quantitative PCR (qPCR) analysis has been used to inhibit amplification of both extracellular DNA and DNA in dead or membrane-compromised bacterial cells^12^. Gensberger *et al*.^13^ evaluated DNA-qPCR and PMA-qPCR assays for evaluating microbial water quality. These authors reported that PMA-qPCR compared to DNA-qPCR resulted in higher sensitivity and specificity in detecting *Escherichia coli*, *Enterococcus* spp. and *Pseudomonas aeruginosa*, as well as coliforms (Enterobacteriaceae family), which are indictor species/groups for water microbial quality control.

Several recent studies^10, 11, 14^ have combined high-throughput sequencing and PMA-based methodologies to detect live cells in human and environmental samples. Despite its advantages, the PMA-based approach has known practical and theoretical limitations. For example, the incubation temperature and duration, as well as the concentration of PMA used need to be optimized based on the levels of suspended solids and microbial biomass in the water. These optimizations are based on trial and error, which is costly and time consuming to determine the correct combination of all the abovementioned factors in order to generate reproducible, sensitive and accurate data^15^. Additionally, the technique is also known to lead to false-positive signals due to the penetration of PMA via damaged cell membranes of live microorganisms^12, 15^.

Compared to DNA, RNA degrades more rapidly in the environment^16, 17^. The estimated turnover time or half-lives of prokaryotic RNA is about few minutes. For example the half-lives of *E. coli* RNA is around 5 min and that of *Bacillus subtilis* ranges from 7 to 15 min^18, 19^. Thus, RNA might be a more suitable target for studying live members of water microbial community. To our knowledge there has been no comparison between DNA-, PMA-, and RNA-based 16S rRNA sequencing for detection of live bacterial cells in water samples. The objective of this study was to compare these three techniques for detection of live bacteria in water samples experimentally spiked with different combination of known bacteria or obtained from different sources. Water source characteristics included ultra-pure HPLC-grade water spiked with gram-positive, gram-negative and acid-fast bacterial strains; treated drinking water that showed negative or positive for total coliforms; as well as lake and river water that showed positive for total coliforms and had relatively small and large sediment load, respectively.

## Results and Discussion

**Figure 1:**
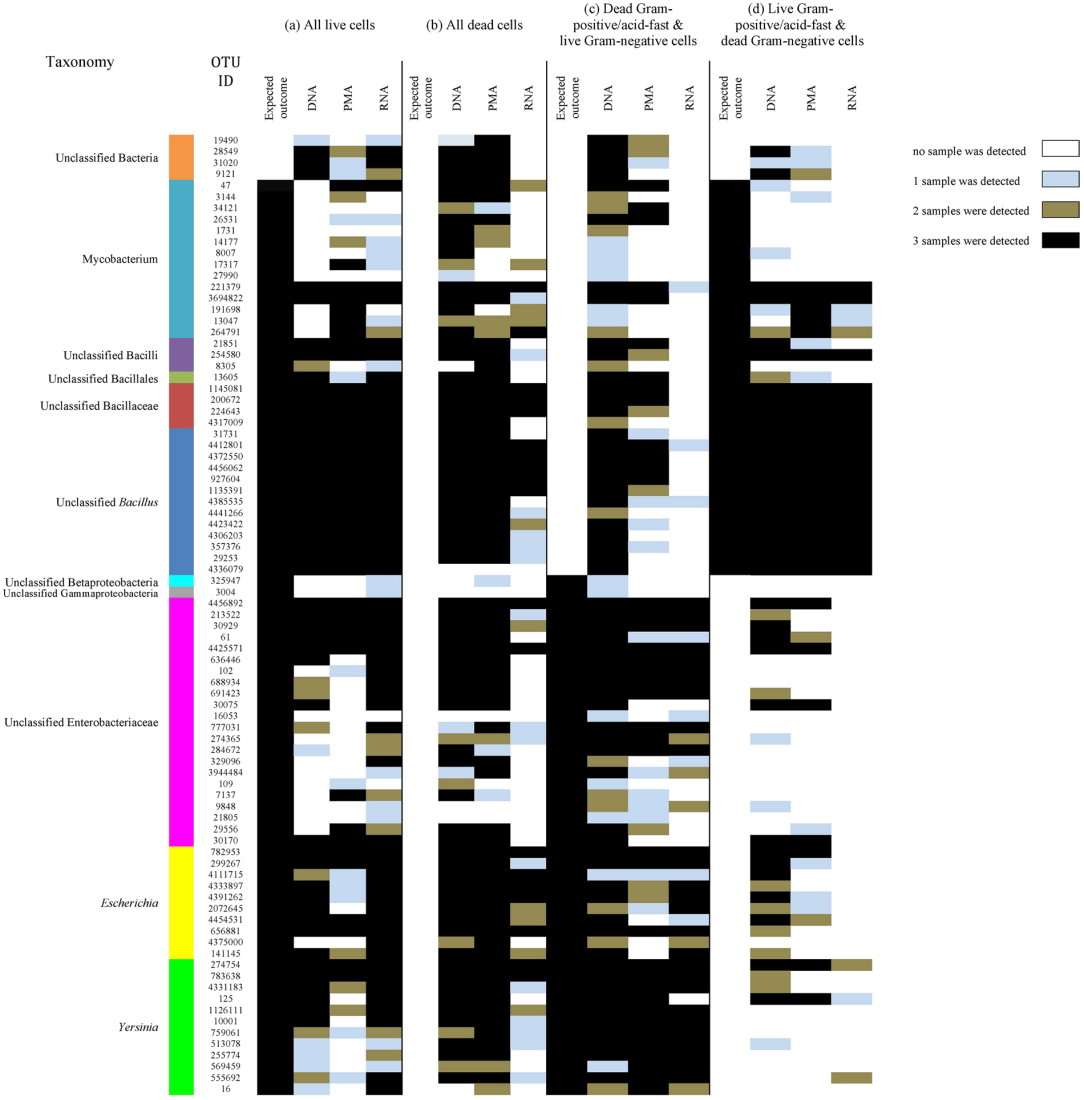
OTU compositions of mini-microbial communities in spiked water samples consisting of (**a**) all live cells of *Bacillus amyloliquefaciens*, *Mycobacterium smegmatis*, *Escherichia coli* and *Yersinia enterocolitica*; (**b**) all dead cells of the aforementioned species; (**c**) dead cells of *B. amyloliquefaciens* and *M. smegmatis*, and live cells of *E. coli* and *Y. enterocolitica;* (**d**) live cells of *B. amyloliquefaciens* and *M. smegmatis*, and dead cells of *E. coli* and *Y. enterocolitica*; determined by the DNA-, RNA-, and PMA-based 16S rRNA MiSeq Illumina sequencing.

**Figure 2:**
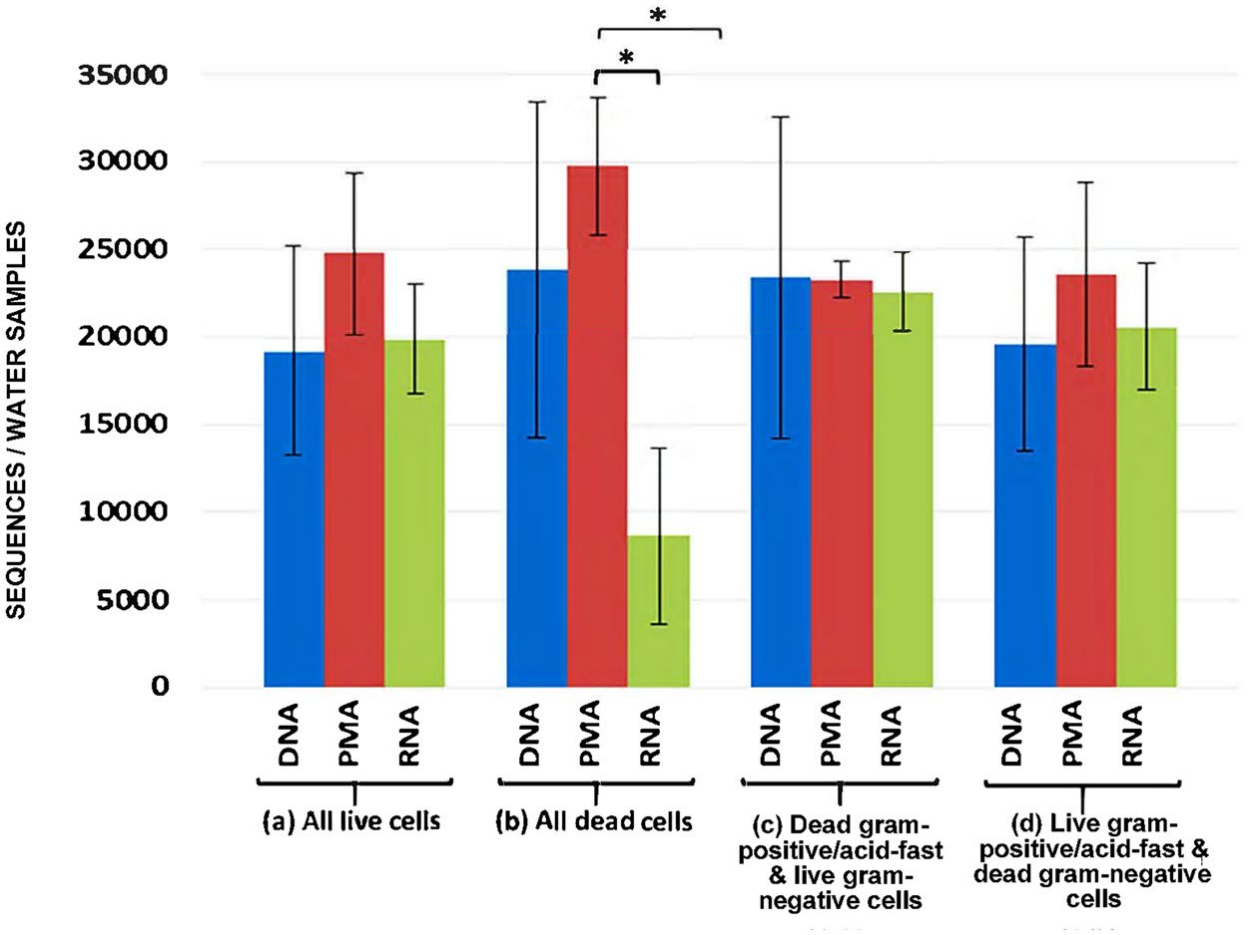
Average sequences in mini-microbial communities of spiked water consisting of (**a**) all live cells of *Bacillus amyloliquefaciens*, *Mycobacterium smegmatis*, *Escherichia coli* and *Yersinia enterocolitica*; (**b**) all dead cells of the aforementioned species; (**c**) dead cells of *B. amyloliquefaciens* and *M. smegmatis*, and live cells of *E. coli* and *Y. enterocolitica;* (**d**) live cells of *B. amyloliquefaciens* and *M. smegmatis*, and dead cells of *E. coli* and *Y. enterocolitica*; determined using DNA-, PMA-, and RNA-based 16S rRNA MiSeq Illumina sequencing. *P < 0.05.

**Figure 3:**
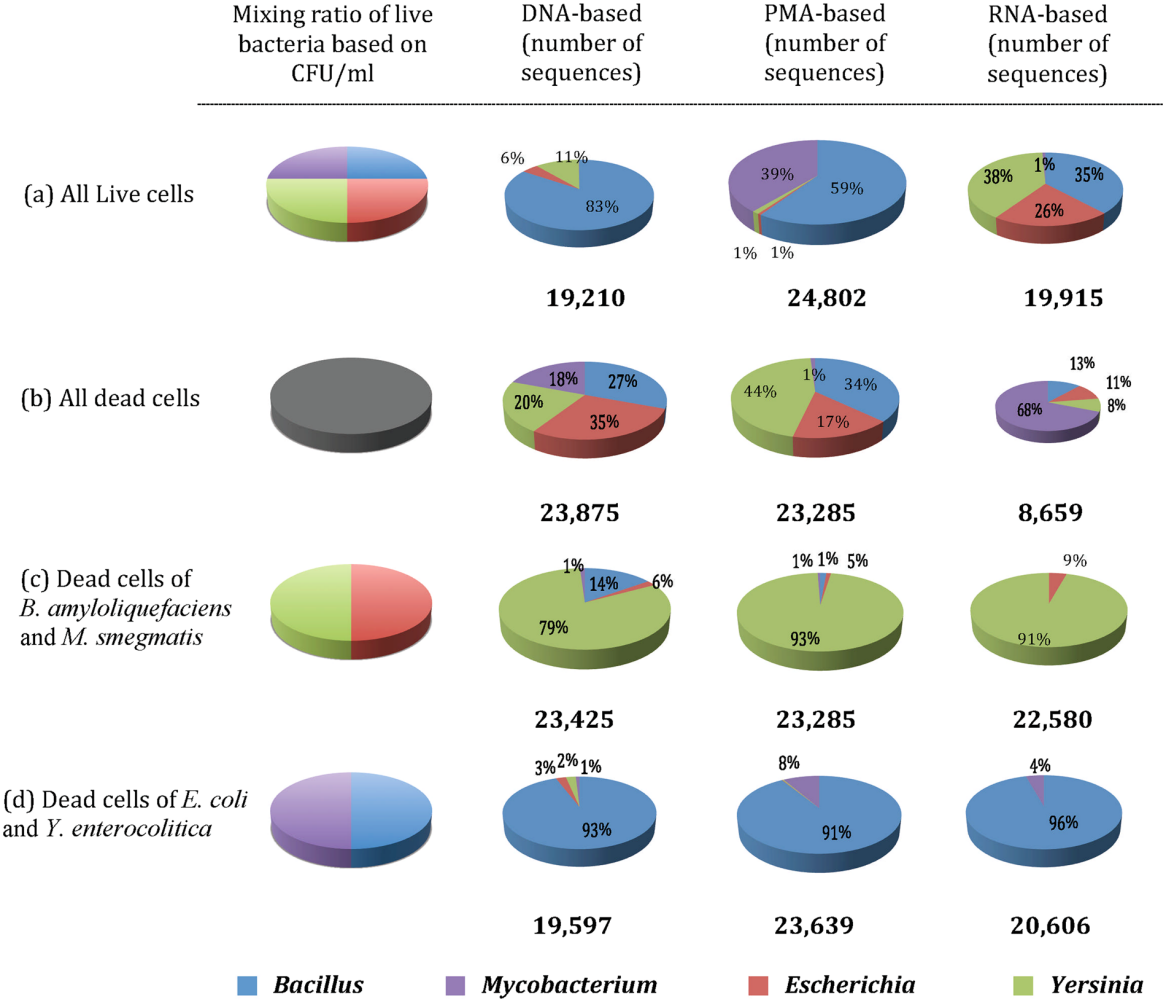
Compositions of mini-microbial communities of spiked water consisting of (**a**) all live cells of *Bacillus amyloliquefaciens*, *Mycobacterium smegmatis*, *Escherichia coli* and *Yersinia enterocolitica*; (**b**) all dead cells of the aforementioned species; (**c**) dead cells of *B. amyloliquefaciens* and *M. smegmatis*, and live cells of *E. coli* and *Y. enterocolitica;* (**d**) live cells of *B. amyloliquefaciens* and *M. smegmatis*, and dead cells of *E. coli* and *Y. enterocolitica*; determined using DNA-, PMA-, and RNA-based 16S rRNA MiSeq Illumina sequencing.

**Figure 4:**
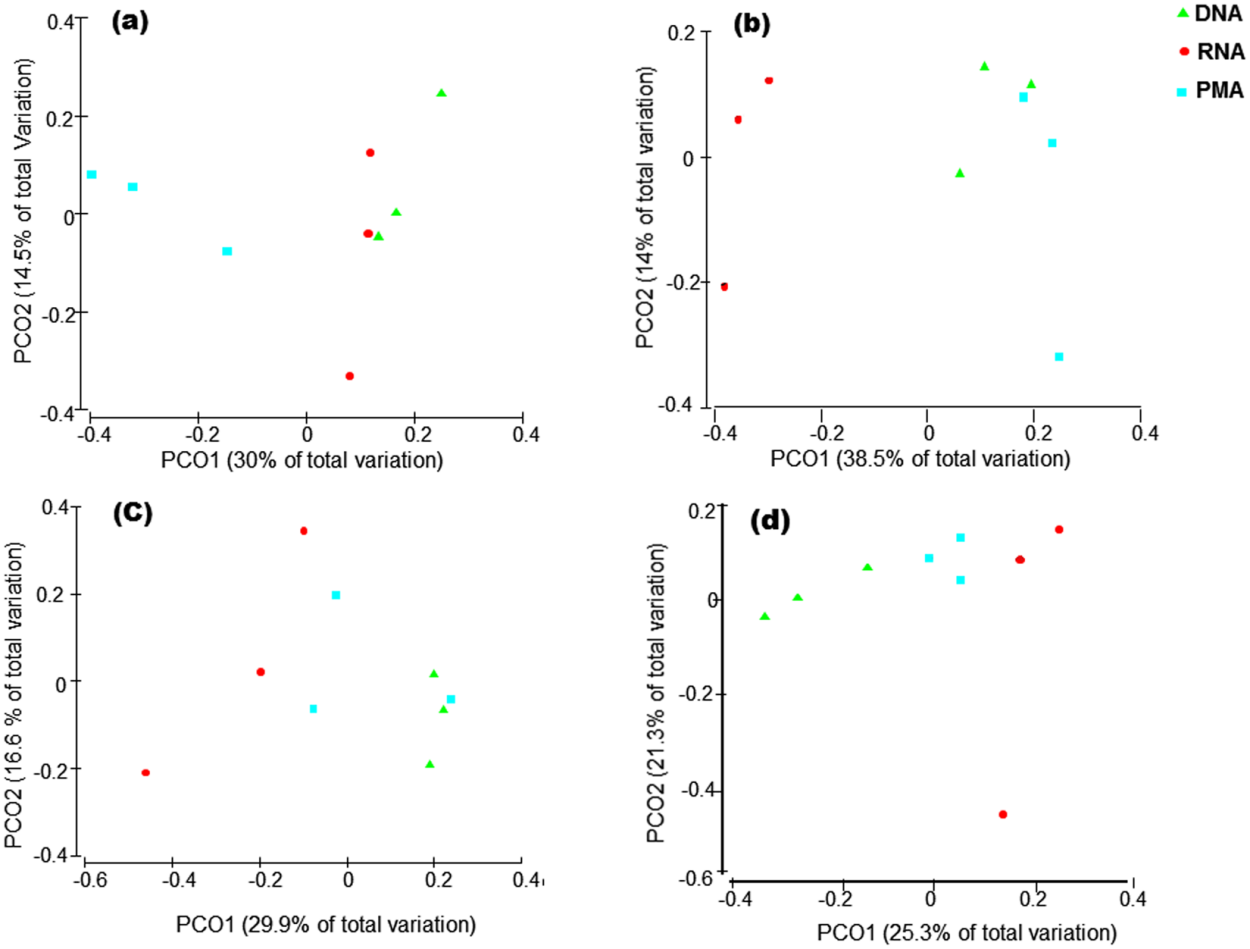
Principal coordinates analysis (PCoA) of unweighted UniFrac distances comparing the β-diversity of mini-microbial communities of spiked water among DNA-, PMA-, RNA-based methods. The mini-microbial communities compositions were as follow: (**a**) all live cells of *Bacillus amyloliquefaciens*, *Mycobacterium smegmatis*, *Escherichia coli* and *Yersinia enterocolitica*; (**b**) all dead cells of the aforementioned species; (**c**) dead cells of *B. amyloliquefaciens* and *M. smegmatis*, and live cells of *E. coli* and *Y. enterocolitica*; (**d**) live cells of *B. amyloliquefaciens* and *M. smegmatis*, and dead cells of *E. coli* and *Y. enterocolitica*; determined using DNA-, PMA-, and RNA-based 16S rRNA MiSeq Illumina sequencing.

Taxonomic classification of clustered OTUs in mini-microbial communities of spiked water samples determined using DNA-, PMA- and RNA-based methods are presented in Figs 1, 2 and 3. We also further used PCoA and PERMANOVA analyses to visualize and compare the variations in β-diversity among mini-microbial communities (Fig. 4; Supplementary Table S1).

Each of the three methods produced results that were different from the original mixing proportions of all live, all dead, or live and dead combinations of bacteria (Fig. 3). In our mixture design experiment, similar to this type of study^20^, the assumption was that the proportion of sequenced OTUs should only depend on the relative proportion of spiked cells into the mixture and the differences between the DNA-, PMA- and RNA-based methodologies, such as the efficiency of DNA vs. RNA extraction, the effect of PMA on the DNA extraction outcome, and the efficiency of RNA to cDNA conversion. However, this assumption was not completely correct as additional bias can be introduced due to other factors. Brooks *et al*.^21^ conducted a set of mixture design experiments during which seven spiked species were either equally mixed first followed by DNA extraction, PCR amplification and high-throughput sequencing, or separately DNA extracted and the DNA was equally mixed for PCR amplification followed by sequencing, or DNA was separately extracted and PCR was separately performed and the PCR products were equally mixed for high-throughput sequencing. The authors reported that mini-microbial community compositions varied among the three experiments with highest similarity to the actual mixing proportions of cells when DNA extractions and PCR amplifications were performed separately on each individual bacterium. The authors concluded that the level of bias was highly dependent on the bacterial species in the mix. In other words, a bacterium’s proportion in the mini-microbial community could be overestimated or understimated due to presence of other bacteria. The study indicated that among factors contributing to such bias perhaps cell lysability, gram-negativity, GC content, and differential amplification efficiency of primers for each species were the major contributing factors. The differences in the genome content of species or in their copy numbers of 16S rRNA genes had less impact on the observed bias than the aforementioned factors.

In this context, Fig. 2 indicates that the total sequence numbers obtained from DNA-, PMA- and RNA-based methods were in closer range when all live (19,210 to 24,801), or a mixture of live and dead cells (19,597 to 29,753) were spiked into the samples. In contrast, when all dead cells were spiked, the number of sequences was lower (*P* < 0.001) in the RNA-based method (8,659) compared to DNA- (23,875) and PMA-based (29,753) methods. The high number of sequences detected by DNA- and PMA-based methods when all dead cells were spiked indicates that these methodologies are not efficient for differentiating between live and dead cells. The RNA-based method, which involved the extraction of microbial cellular RNA from metabolically active cells following by its conversion to cDNA and sequencing, while was superior for detection of live bacterial cells still identified a considerable number of 16S rRNA targets from samples spiked with dead cells (Fig. 3b). Figure 3b clearly shows that the majority (68%) of detected 16S rRNA sequences using RNA-based method belonged to *M. smegmatis*, which similar to other mycobacteria has a cell wall structure that is more rigid than other bacterial species^22^. As a result, we speculate that detected 16S rRNA targets using RNA-based method (Fig. 3b) was perhaps due to inefficiency of heat treatment step for complete breakdown and inactivation of *M. smegmatis* cells leaving a proportion of cells to be metabolically active. The DNA- and PMA-based analyses of these samples further supports this hypothesis (Fig. 3b, Supplementary Table S1). Using DNA-based approach all 4 dead spiked species were identified in the community with the lowest proportion for *Mycobacterium* (18%) and the highest for *Escherichia* (35%) suggesting that when cells were properly disrupted (heat exposure followed by chemical and mechanical lysis during the extraction procedures) and DNA is exposed, DNA-based methods can amplify the DNA irrespective of its source (live or dead cells). In contrast, when PMA-based method was employed to assess the mini-microbial community of spiked water with all dead cells, *Escherichia, Bacillus*, and *Yersinia* were highly amplified ranging from 22% to 44% of the sequences, while *Mycobacterial* proportion was only 1%. The results firstly indicated that PMA had low efficiency for removal of DNA from dead cells when microbial biomass was high, and secondly suggested that a large proportion of *Mycobacterial* cells – compared to other species in the mix – were perhaps still intact, which prevented PMA from penetration and binding to DNA.

Our results are supported with previous observations showing that PMA treated DNA from dead gram-negative *E. coli* cells were still detectable by qPCR as the DNA from dead/membrane-compromised cells were not completely removed by PMA treatment^23^. Authors articulated that the levels of biomass in water samples may interfere with the ability of the PMA-qPCR method to detect live cells. Varma *et al*.^12^ similarly showed that both gram-positive and gram-negative bacterial DNA present in wastewater can be simultaneously detected by qPCR after treatment with PMA when the levels of biomass were high.

Comparison of the prescribed proportions of bacteria with the results of the DNA-based sequencing method when all live bacteria were spiked can be used to evaluate whether the observed proportions of bacteria were overestimated or underestimated by the presence of other bacteria in the mini-microbial community (Fig. 3a). Our results showed that the sequence reads obtained from mini-microbial community were dominated by *Bacillus* (83% of the community) followed by *Yersinia* (11%) and *Escherichia* (6%), whereas *Mycobacterium* was almost undetectable in the community. Perhaps, this can be explained by differences in the copy number of 16S rRNA molecule per cell among species; for instance, the acid-fast *M. smegmatis* have only 2 copies of 16S rRNA molecule whereas *B. amyloliquefaciens* has 10 copies. The gram-negative strains *E. coli* and *Y. enterocolitica* have similar 16S rRNA copy number of 7 (Ribosomal Database Project [http://rdp.cme.msu.edu]). Moreover, the underestimation of *Mycobacterium* perhaps is due to low lysability of this bacterium when only exposed to chemical and mechanical cell disruption methods. In addition, among the 4 spiked species, *Mycobacterium* has the highest GC content (65.6%)^24^, which further contributes to low amplification efficiency of this bacterium. In contrast, *Bacillus* had the lowest GC content (42%)^25^ followed by *Yersinia (*47%)^26^, and *Escherichia* (50%)^27^. Our results were showing the same trend (Fig. 3a) and supported that species with low GC content are more amplifiable and thus are promoted during the PCR amplification. We should also take into account that although spiked cells were harvested from their late log cultures they may still contain dead cells that could be amplified using DNA-based method. This should be the major reason for the observed differences between the DNA- and RNA-based methods, as RNA-based method only amplified the live and metabolically active proportion of mixed bacterial cells (Fig. 3a, Supplementary Table S1).

The pattern observed during PMA-based amplification of the same community (all live spiked) was very different from both DNA- and RNA-based methods (Fig. 3a and Supplementary Table S1). As indicated above the spiked water with live bacteria may have contained a number of dead cells or cells with damaged/compromised membranes due to osmotic stress when spiked into HPLC water^10, 28^ or heating and light exposure during PMA treatment^29^. This could have facilitated the penetration of PMA into such cells, and hence, excluding them as live cells. Hellein *et al*.^30^ in spiked environmental waters tested with PMA by qPCR reported that PMA can penetrate into membrane-compromised bacteria even though they are live. The abovementioned stressors perhaps may sensitize bacterial cells especially those of gram-negative bacteria resulting in suppression of these species in the sequenced mini-microbial community of all live spiked cells.

The above-mentioned mechanisms also explain the observed patterns of microbiota when a combination of live and dead cells was spiked. Regardless of Gram status of spiked cells, RNA-based method was superior to DNA- and PMA-based methods in distinguishing live cells although the proportion of these cells were over- or underestimated (Fig﻿. 1, Fig. 3c,d, Supplementary Table S1). For spiked water with live gram-negative and dead gram-positive/acid-fast bacteria, the RNA-based method did not detect the dead gram-positive bacteria, while DNA- and PMA-based methods did (15% in DNA-based and 2% in PMA-based; Fig. 3c). However, the proportion of *Yersinia* was overestimated (91%) and *Escherichia* was underestimated (9%) in the community. This might be in one hand due to the higher GC content of *Escherichia* and reduced amplification efficiency for this species^31^, but more importantly due to misclassification of OTUs associated with these two species due to the similarity of 16S rRNA in members of Enterobacteriaceae family^32^. Similarly, for spiked water with live gram-positive/acid-fast and dead gram-negative bacteria, the RNA-based method did not detect the dead gram-negative species but overestimated *Bacillus* proportion (96%) and underestimated *Mycobacterium* (4%) most likely due to lower GC content of *Bacillus*. Compared to RNA-, DNA- and PMA-based methods estimated that 5% and 1% of the community to be associated with dead species, respectively (Figs 1 and 3d). Other studies^12, 23, 29, 33^ have similarly reported that the DNA from dead/membrane-compromised gram-negative and/or gram-positive cells were not completely removed by PMA treatment, and therefore detected by qPCR analysis.

**Figure 5:**
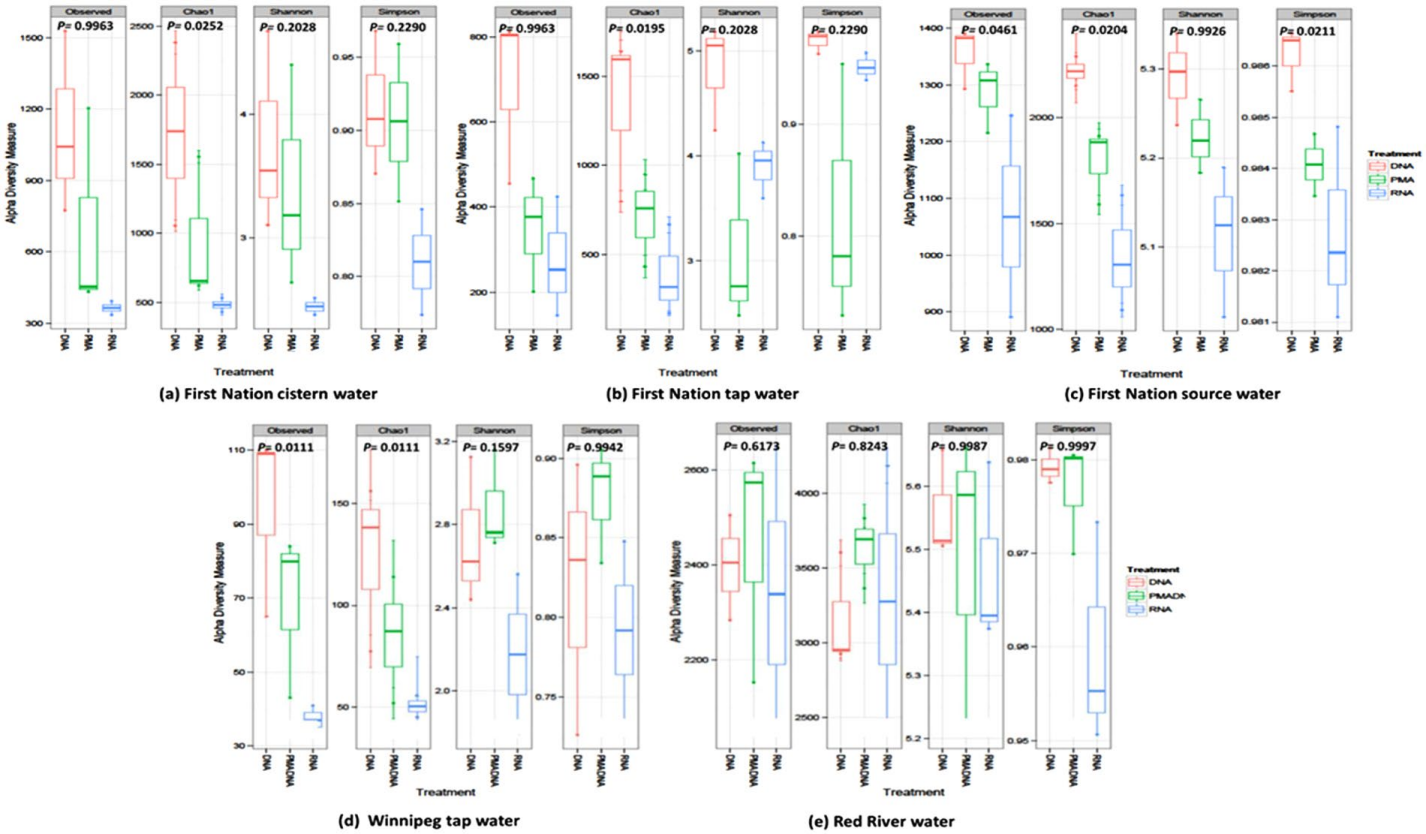
α-diversity indices of microbiota of water samples collected from different sources and assessed by DNA-, PMA, and RNA-based 16S rRNA MiSeq Illumina sequencing.

For the five environmentally relevant water sources, Illumina paired-end sequencing generated on average 27,727 of high quality sequences per sample. An even depth of 11,000 sequences per sample was used for comparison of α-diversity measures among water sources and DNA-, PMA- and RNA-based methods (Fig. 5). Regardless of water source, the Chao1 richness of bacterial community was highest for the DNA-based approach and lowest for RNA-based approach (*P* < 0.05), with the exception of red river water. The α-diversity metrics differ in the way they account for rare and abundant species. In case of Chao1, it estimates the number of undiscovered species, and as DNA-based method does not differentiate between live and dead bacteria, it identifies more OTUs and thus overestimates the community richness.

**Figure 6:**
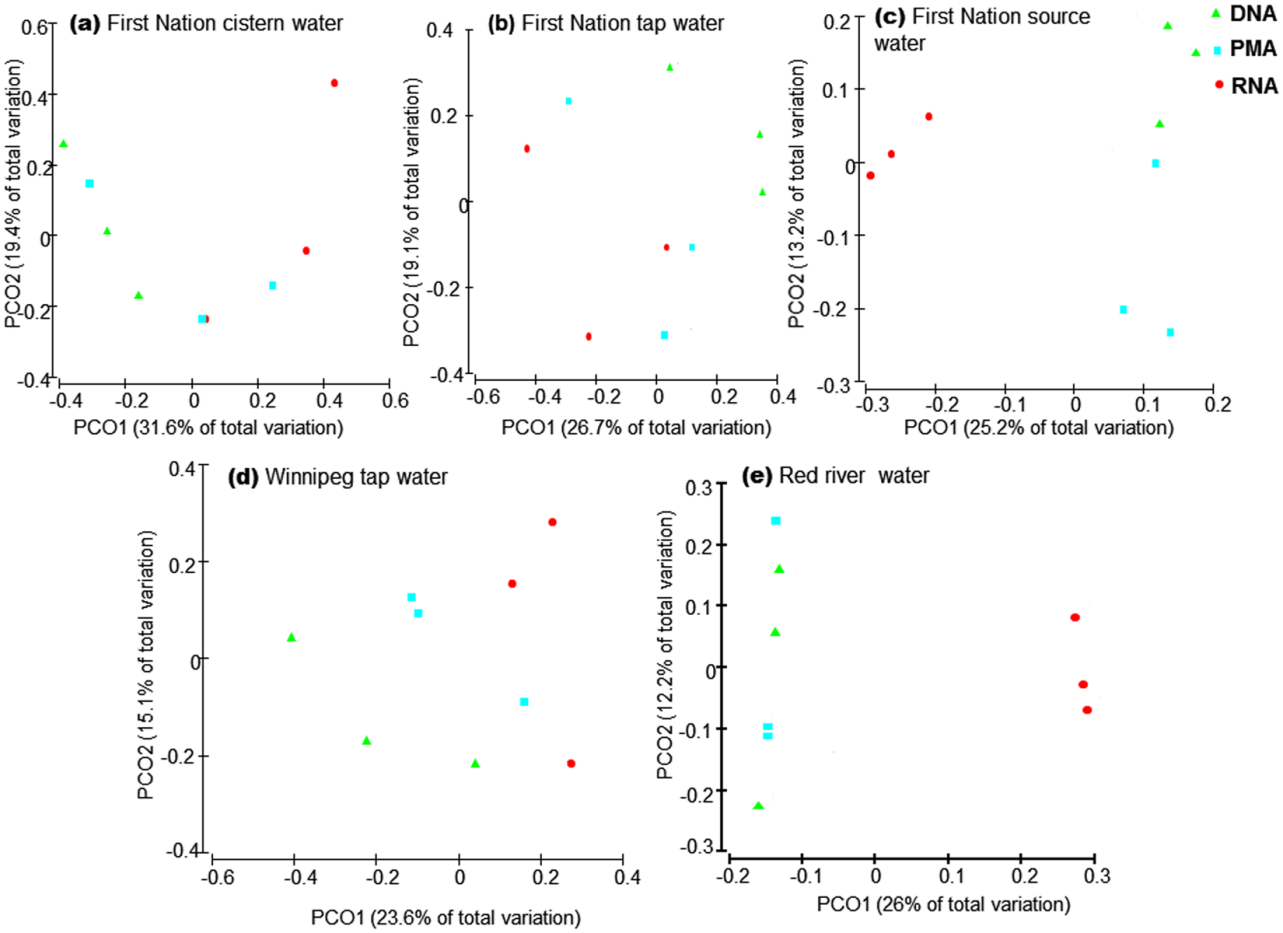
Principal coordinates analysis (PCoA) of unweighted UniFrac distances of water samples collected from different sources to compare microbiota β-diversity between DNA-, PMA-, and RNA-based 16S rRNA MiSeq Illumina sequencing.

The β-diversity among water sources and DNA-, PMA- and RNA-based methods were visualized and compared using PCoA and PERMANOVA (Fig. 6, Supplementary Table S2). The RNA-based method separated from the DNA- and PMA-based methods particularly in lake and river water that contained proportionally larger amounts of live and dead bacterial cells than the treated water.

**Figure 7:**
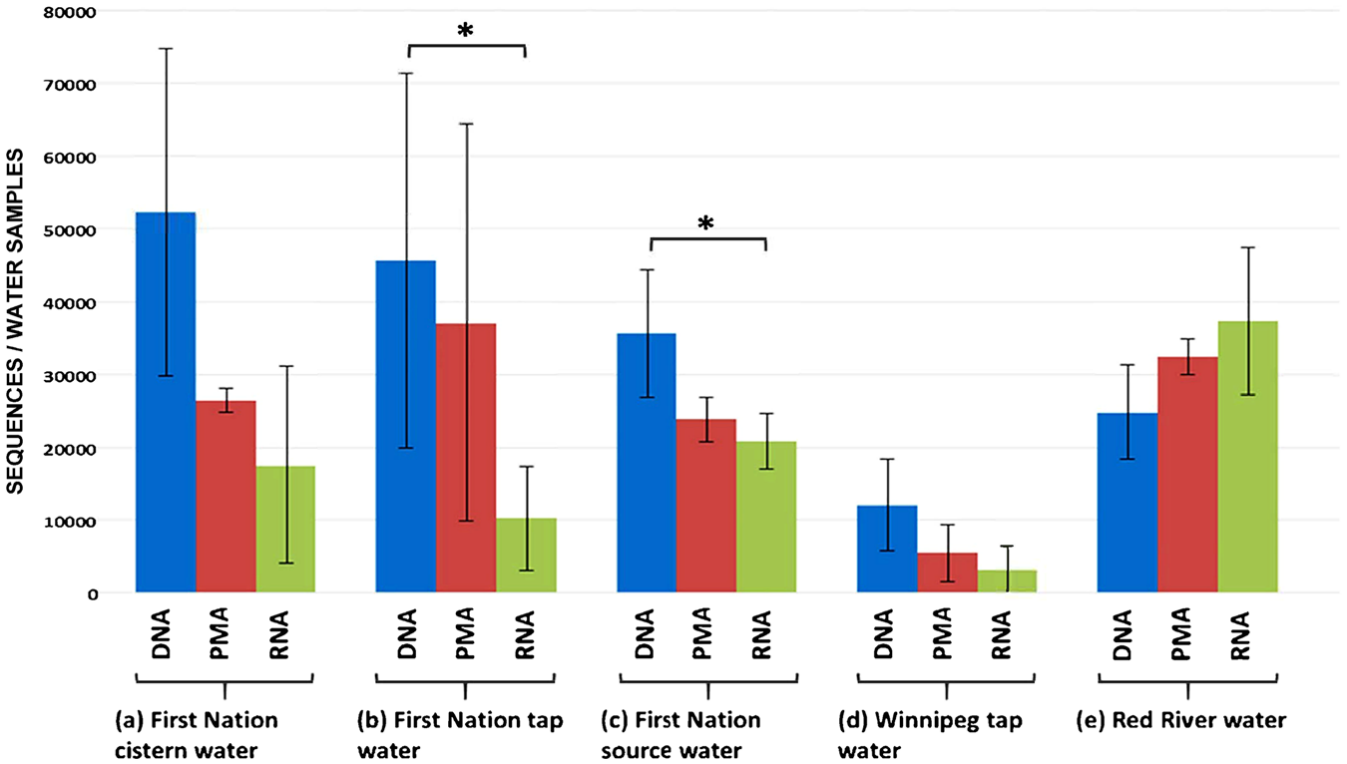
Average number of sequences in water samples collected from different sources analyzed using DNA-, PMA-, and RNA-based 16S rRNA MiSeq Illumina sequencing. *P < 0.05.

**Figure 8:**
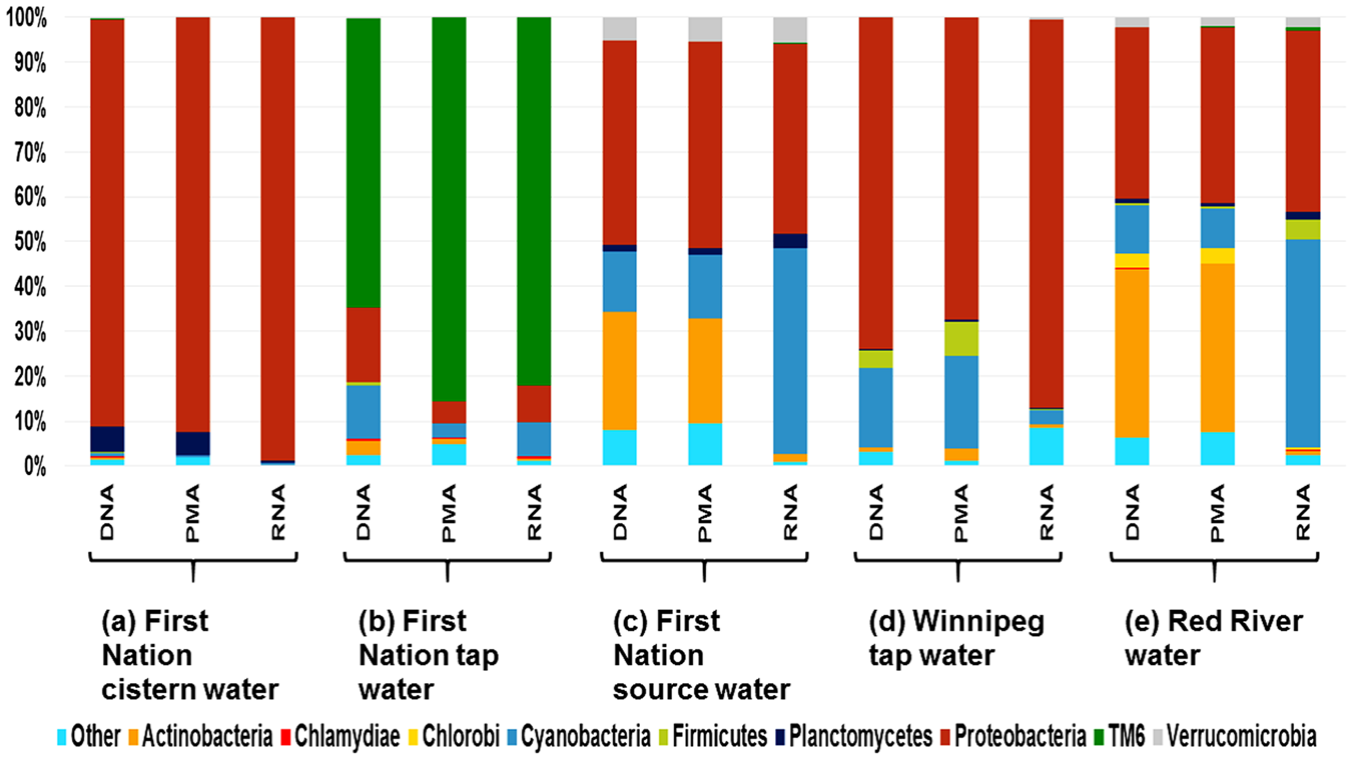
Microbiota compositions in water samples collected from different sources determined using DNA-, PMA-, and RNA-based 16S rRNA MiSeq Illumina sequencing.

The average numbers of sequences in five environmentally relevant water sources are presented in Fig. 7. The taxonomic classification of clustered OTUs for these samples revealed the presence of 47 bacterial phyla. Phyla with abundances greater than 0.1% of the community were used to compare the microbiota composition generated using DNA-, PMA-, and RNA-based methods (Fig. 8). The DNA- and PMA-based methods showed a close agreement in phyla, whereas the RNA-based method showed pronounced differences from the DNA- and PMA-based methods for the lake and river samples, and the treated water collected in the water treatment plant (Fig. 8).

As indicated earlier optimization of PMA-based sequencing for capturing live members of microbial community in water samples has major difficulties. Different proportions of gram-negative, gram-positive and acid-fast bacteria are present within each community at different physiological growth stages and PMA reaction with DNA from these communities differ. Several studies^12, 23, 29, 33^ reported that PMA-based qPCR may be less effective for samples with a complex mixture of gram-positive and gram-negative bacterial cell, or when the ratio of dead to live cells was high.

Our study supports these observations and recommends RNA-based approach as a superior tool for proper estimation of live (either dormant or metabolically active) members of bacterial community in the water. That being said, it should be noted that RNA is less stable than DNA and might be more difficult to purify from certain environmental samples^34–36^. Our data also recognizes that the abundances of the community members are always overestimated or underestimated due to biases introduced during the DNA or RNA extraction steps (e.g. due to lisability of cells and Gram status) and differential amplification of target during PCR (e.g. due to GC content and primer efficiency). These biases will continue to be a challenge for microbial community analyses regardless of DNA-based or RNA-based 16S rRNA sequencing method of choice.

## Materials and Methods

### Bacterial strains and culture condition used for spiking water

Gram-positive *Bacillus amyloliquefaciens* (strain BS6), acid-fast *Mycobacterium smegmatis* and two gram-negative strains, *Escherichia coli* and *Yersinia enterocolitica*, obtained from the Microbiome Laboratory (Department of Animal Science, University of Manitoba, Canada) were cultured in the liquid Luria-Bertani media (LB; Difco, Fisher Scientific, Edmonton, AB, Canada) on a rotary shaker (New Brunswick Scientific, Edison, NJ, USA). *E. coli* and *Y. enterocolitica* were cultured at 200 rpm and 37 °C for 16–18 h. *M. smegmatis* was cultured at 200 rpm and 37 °C for 72 h. *B. amyloliquefaciens* was cultured at 180 rpm and 30 °C for 16 to 18 h. All bacteria were collected from late log cultures to minimize the number of dead bacteria within the cultures.

For each of the cultured species, half of the culture was killed by placing flasks in a water bath at 95 °C for 30 min and confirming that aliquots (100 μl) did not produce any visible colonies on the LB agar (Difco) upon incubation. To experimentally create mini-microbial communities, ultra-pure HPLC-grade (Fisher Scientific, Ottawa, ON, Canada) was used as the water source and spiked with mixtures of bacterial species present at a concentration of 1.5 × 10^4^ CFU/ml in triplicate as follow: (1) four live bacterial species, (2) four dead bacterial species, (3) dead gram-positive and acid-fast species plus live gram-negative species, (4) dead gram-negative species plus live gram-positive and acid-fast species, and (5) HPLC grade water without any bacterial culture (negative control).

### Environmental water sample collection

Water samples (n = 3 per treatment group) were collected from a First Nation community in Northern Manitoba in July 2014, and in the City of Winnipeg in September 2014 using standard methods^37^: SM9060A (sample bottle pretreatment) and SM 9060B (preservation and storage). Water samples in the Northern community included: 1) tap water from homes that are served by piped water from the water treatment plant (0–3 CFU/100 ml total coliforms, and 0–2 CFU/100 ml *E. coli*), 2) tap water from homes which receive potable water delivered from the water treatment plant by a water truck to a cistern (water holding tank) (2–430 CFU/100 ml total coliforms, and 1–400 CFU/100 ml *E. coli*), and 3) samples collected from the lake that is the source water to the water treatment plant (50–690 CFU/100 ml total coliforms, and 0–330 CFU/100 ml *E. coli*). Samples were transported in coolers to Winnipeg by air on the same day of collection, or in the next morning after stored overnight in a refrigerator. Water samples in the City of Winnipeg included 1) tap water from homes that are served by piped water from the City’s water treatment plant (0 CFU/100 ml total coliforms or *E. coli*), and 2) samples collected from the Red River flowing through the city (27,000–37,000 CFU/100 ml total coliforms, and 0–1,000 CFU/100 ml *E. coli*).

All collected samples were immediately processed upon receiving in the level 2 biological safety Microbiome Laboratory (Department of Animal Science, University of Manitoba, Canada). This processing included determinations of total coliform and *E. coli* counts using the standard membrane filter procedure as outlined in SM9222^37^. Briefly, 100 ml of water sample was filtered through a sterile 0.20 µm Polyethersulfone membrane filter (Mo Bio Laboratories, Inc. West Carlsbad, CA, USA). The filter paper was placed on agar plates containing chromogenic Brilliant *E. coli* and coliform medium (Fisher Scientific, Ottawa, ON, Canada) and incubated at 35 °C for 24 h. Some samples required dilution as bacterial counts were too high.

### Extraction of DNA and RNA from water samples and DNA from PMA treated water

For the HPLC graded water spiked with mixtures of bacterial species, aliquots of 100 ml spiked water were filtered through 0.20 μm Polyethersulfone filter (Mo Bio Laboratories Inc., West Calsbad, CA, USA). For the environmental water samples, 500 ml aliquots were filtered through 0.20 μm Polyethersulfone filter (Mo Bio). DNA and RNA were extracted using Powerwater DNA and RNA isolation kits (Mo Bio), respectively, that included a bead-beating step for mechanical lysis of bacteria. *DNase I* treatment step was included during RNA extraction for removal of DNA. RNA was transcripted to cDNA using a reverse transcription kit (QuantiTec, Qiagen Scientific, Germantown, MD, USA). For the PMA treatment, a modified method based on Hellein *et al*.^30^ and Nocker *et al*.^10^ was used. In brief, following filtering of 500 ml aliquots of water, 5 μl PMA was added onto the filter when 2 ml water was left, to give a final PMA concentration of 50 μm (PMA dye, Biotium, Inc., Hayward, CA, USA). The PMA spread evenly across the filter. The filters were incubated on a rocker with aluminum foil for 20 min and then placed on an ice block set 20 cm from a light source. They were exposed for 15 min to 500 W Halogen light to cross-link the PMA to DNA. DNA was extracted immediately after PMA treatment using MoBio Powerwater DNA kit. Triplicate samples were processed for each defined mixture of spiked water. The quantity of the DNA and cDNA were measured using NanoDrop 2000 spectrophotometer (Thermo Scientific, Waltham, MA, USA). The quality was measured by PCR amplification of 16S rRNA, using forward primer 27 F (AGAGTTTGATCMTGGCTCAG) and reverse primer 342 R (CTGCTGCSYCCCGTAG), after DNA and cDNA were normalized to the concentration of 10 ng/μl^38^.

### Library construction and Illumina sequencing

A library of V4 region of 16S rRNA was constructed using modified F515 and R806 primers^39^ as described previously^40^. PCR reaction for each sample was performed in duplicate and contained 1.0 µl of pre-normalized DNA, 1.0 µl of each forward and reverse primers (10 µM), 12 µl HPLC grade water (Fisher Scientific, Ottawa, ON, Canada) and 10 µl 5 Prime Hot MasterMix (5 Prime, Inc., Gaithersburg, MD, USA). Reactions consisted of an initial denaturing step at 94 °C for 3 min, followed by 35 amplification cycles at 94 °C for 45 sec, 50 °C for 60 sec, and 72 °C for 90 sec; finalized by an extension step at 72 °C for 10 min in an Eppendorf Mastercycler pro (Eppendorf, Hamburg, Germany). Next, PCR products were purified using ZR-96 DNA Clean-up Kit (ZYMO Research, Irvine, CA, USA) to remove primers, dNTPs and reaction components. The V4 library was then generated by pooling 200 ng of each sample, quantified by Picogreen dsDNA (Invitrogen, Burlington, NY, USA). The pooled library was diluted to a final concentration of 5 pM using pre-chilled hybridization buffer (HT1) (Illumina, Irvine, CA, USA) measured by Qubit 2.0 Fluorometer (Life technologies, Ottawa, ON, Canada). In the final step, 15% of PhiX control library was spiked into the amplicon library to improve the unbalanced and biased base composition. For Illumina sequencing, customized sequencing primers for read1 (5′-TATGGTAATTGTGTGCCAGCMGCCGCGGTAA-3′), read2 (5′-AGTCAGTCAGCCGGACTACHVGGGTWTCTAAT-3′) and index read (5′-ATTAGAWACCCBDGTAGTCCGGCTGACTGACT-3′), and mixture of sample and PhiX library were added to the MiSeq Reagent Kit V2 (300-cycle; Illumina, San Diego, CA, USA). The 150 paired-end sequencing reaction was performed on a MiSeq Illumina platform at the Microbiome Laboratory (Department of Animal Science, University of Manitoba, Canada). The sequence data are uploaded into the Sequence Read Archive (SRA) or NCBI (http://www.ncbi.nlm.nih.gov/sra) and accessible through accession number SRR2983316.

### Bioinformatics and statistical analysis

The FLASH assembler^41^ was used to merge overlapping paired-end Illumina fastq files. All the sequences with mismatches or ambiguous calls in the overlapping region were discarded. The output of fastq file was then analyzed by downstream computational QIIME pipelines^42^. Assembled reads were de-multiplexed according to barcode sequences, chimeric reads were filtered using UCHIME^43^ and sequences were assigned to Operational Taxonomic Units (OTU) using the QIIME implementation of UCLUST^44^ at 97% pairwise identity threshold. Taxonomies were classified to the representative sequence of each OTU using RDP classifier^45^ and aligned with the Greengenes Core reference database^46^ using PyNAST algorithms^47^. A phylogenetic tree was built with FastTree 2.1.3.^48^ for further comparisons between microbial communities.

Within-community diversity (α-diversity) was calculated by different indices of species richness and evenness including Shannon, Simpson, Chao1, and observed number of species using the open source software QIIME. Permutational analysis of variance (PERMANOVA, Anderson, 2005) and principal coordinates analysis (PCoA) of unweighted UniFrac distance matrices^49^ were used to assess between-sample differences in microbiota diversity and community structures (Warwick and Clarke, 2006). The differences of α-diversity indices and number of sequences among treatments were tested using MIXED procedure (Tukey studentized range adjustment) of SAS (version 9.3, SAS Institute Inc., Cary, NC, USA) with the effect of sequencing method as the fixed factor. For non-normally distributed data, GLIMMIX procedure of SAS fitted with Poisson or negative binomial distributions was used. The differences between treatments were considered significant at *P* < 0.05.

## Electronic supplementary material

Supplementary_Tables
