# Author Correction: Comparison of DNA-, PMA-, and RNA-based 16S rRNA Illumina sequencing for detection of live bacteria in water

**DOI:** 10.1038/s41598-018-35437-w

**Published:** 2018-11-22

**Authors:** Ru Li, Hein Min Tun, Musarrat Jahan, Zhengxiao Zhang, Ayush Kumar, W. G. Dilantha Fernando, Annemieke Farenhorst, Ehsan Khafipour

**Affiliations:** 1 Department of Soil Science, University of Manitoba, Winnipeg, MB R3T 2N2 Canada; 2 Department of Animal Science, University of Manitoba, Winnipeg, MB R3T 2N2 Canada; 3 Department of Medical Microbiology, University of Manitoba, Winnipeg, MB R3T 2N2 Canada; 4 Department of Microbiology, University of Manitoba, Winnipeg, MB R3T 2N2 Canada; 5 Department of Plant Science, University of Manitoba, Winnipeg, MB R3T 2N2 Canada; 6 Present Address: Department of plant protection, Yunnan Agricultural University, Kunming, Yunnan province 650201 China; 7 Present Address: Department of Pediatrics, University of Alberta, Edmonton, AB Canada

Correction to: *Scientific Reports* 10.1038/s41598-017-02516-3, published online 18 July 2017

The original version of this Article contained a typographical error in the spelling of the author W.G. Dilantha Fernando, which was incorrectly given as Dilantha Fernando.

This error has now been corrected in the PDF and HTML versions of the Article, as well as the Supplementary Information file that accompanies the Article.

