# Supplementary material for: Comparison of DNA-, PMA-, and RNA-based 16S rRNA Illumina sequencing for detection of live bacteria in water
Source: Sci Rep. 2017 Jul 18;7:5752. doi: 10.1038/s41598-017-02516-3 (PMC5515937; doi:10.1038/s41598-017-02516-3)
Supplement: Supplementary file 1 — Supplementary_Tables [file 41598_2017_2516_MOESM1_ESM.pdf]

Comparison of DNA-, PMA-, and RNA-based 16S rRNA Illumina sequencing for detection of live bacteria in water

Ru Li[1,†,*], Hein Min Tun[2,†,], Musarrat Jahan[1,2,†], Zhengxiao Zhang[2,3], Ayush Kumar[3,4], W. G. Dilantha Fernando[5], Annemieke Farenhorst[1,‡] and Ehsan Khafipour[2, 3, ‡]**

Supplementary Table S1. Permutational Monte Carlo testing of unweighted UniFrac distances of mini-microbial community of spiked water samples with different combination of live and dead bacteria to compare the clustering pattern of mini-microbiota between DNA-, PMA-, and RNA-based methods followed by high-throughput MiSeq Illumina sequencing

Spiked water treatment group	Combination of live and dead bacterial cell used for spiking water samples								Beta-diversity comparison group	*P*-value[1]
	Live cells				Dead cells					
	Gram-positive	Gram-negative		Acid-fast	Gram-positive	Gram-negative		Acid-fast		
	Bacillus amyloliquefaciens	*Escherichia coli*	*Yersinia enterocolitica*	*Mycobacterium smegmatis*	*Bacillus amyloliquefaciens*	*Escherichia coli*	*Yersinia enterocolitica*	*Mycobacterium smegmatis*		
									DNA *vs.* RNA	0.34
1	✔	✔	✔	✔					DNA *vs.* PMA	0.06
									PMA *vs.* RNA	0.14
									DNA *vs.* RNA	0.05
2					✔	✔	✔	✔	DNA *vs.* PMA	0.29
									PMA *vs.* RNA	0.04
									DNA *vs.* RNA	0.09
3		✔	✔		✔			✔	DNA *vs.* PMA	0.37
									PMA *vs.* RNA	0.34
									DNA *vs.* RNA	0.13
4	✔			✔		✔	✔		DNA *vs.* PMA	0.13
									PMA *vs.* RNA	0.26

[1]*P*-values based on Monte Carlo test. The bigger the *P*-values more similarly community membership was characterized between the compared methodologies and vice versa.

Supplementary Table S2. Permutational Monte Carlo testing of unweighted UniFrac distances of different water sources to compare the clustering pattern of microbial communities between DNA-, PMA-, and RNA-based methods followed by high throughput MiSeq Illumina sequencing

Water source	Beta-diversity comparison group	*P*-value[1]
First Nation Source Water	DNA *vs*. PMA	0.37
	DNA *vs*. RNA	0.10
	PMA *vs*. RNA	0.10
First Nation Cistern	DNA *vs*. PMA	0.37
	DNA *vs*. RNA	0.13
	PMA *vs*. RNA	0.45
First Nation Tap	DNA *vs*. PMA	0.26
	DNA *vs*. RNA	0.15
	PMA *vs*. RNA	0.45
Winnipeg Tap	DNA *vs*. PMA	0.33
	DNA *vs*. RNA	0.22
	PMA *vs*. RNA	0.28
Red River	DNA *vs*. PMA	0.61
	DNA *vs*. RNA	0.11
	PMA *vs*. RNA	0.15

[1]*P*-values based on Monte Carlo test. The bigger the *P*-values more similarly community membership was characterized between the compared methodologies and vice versa.
